# Evidence of an increased prevalence of multiple sclerosis: a population-based study of Tehran registry during 1999–2018

**DOI:** 10.1186/s12883-020-01747-8

**Published:** 2020-05-02

**Authors:** Amir Almasi-Hashiani, Mohammad Ali Sahraian, Sharareh Eskandarieh

**Affiliations:** 1 Department of Epidemiology, School of Health, Arak University of Medical Sciences, Arak, Iran; 2 Multiple Sclerosis Research Center, Sina Hospital, Hassan Abad square, Neuroscience Institute, Tehran University of Medical Sciences, Tehran, Iran

**Keywords:** Epidemiology, Iran, Multiple sclerosis, Prevalence, Tehran

## Abstract

**Background:**

The epidemiological characteristics of multiple sclerosis (MS) have been investigated in various studies, which have revealed that the prevalence of MS varies across countries. The present study was conducted to investigate the longitudinal prevalence of MS in Tehran, Iran.

**Methods:**

The present population-based study was conducted in Tehran, the capital of Iran from 1999 to 2018 based on the annual report data provided by the Iranian MS Society (IMSS) registry system. The age-standardized and crude prevalence were estimated using population data presented by the Statistical Centre of Iran.

**Results:**

A total of 21,580 MS cases were registered and included in the analysis. Among the participant patients, 24.99% (5393) and 75.01% (16,187) of cases were male and female, respectively. The mean age of MS onset was 28.8 years (S.D: 8.7). The age-standardized prevalence (ASP) of MS increased from 73.7 (95%CI: 72.1–75.2) per 100,000 people in 2006 to 137.6 (95% CI: 135.7–139.5) per 100,000 people in 2018. The ASP of MS in 2018 was estimated to be 67.9 (95%CI: 66.0–69.8) and 207.3 (95%CI: 204.0–210.7) per 100,000 people among males and females, respectively. The age-standardized female-to-male ratio of MS ranged from 3.7 (in 2010) to 2.06 (in 2017).

**Conclusion:**

The findings of this study suggested that the prevalence of MS in Tehran province is relatively high, and the occurrence of the disease is more common in the age groups under 40 years as compared with older-aged groups. In line with reports provided for various regions of the world, the prevalence of MS was higher among women. Similarly, the findings of this study revealed that the female-to-male ratio was 2.14 in 2018.

## Highlights

- The prevalence of MS is high in Tehran.
- The disease occurrence is more common in the under-40 age group than in the older ones.
- The age standardized female to male ratio of MS decreased from 3.7 (in 2010) to 2.06 (in 2017) in Tehran province.

## Background

Multiple sclerosis (MS) is a chronic autoimmune neurological disease [1], which is recognized as the most prevalent inflammatory neuroimmunological disorder among young adults [2, 3].

The report of 2,221,188 MS cases in the world in 2016 as compared with 1990 represents an increase of over 10%. The highest prevalence of MS was reported for North America, Western Europe, and Australia, while the lowest rate was related to Sub-Saharan Africa, Central Africa, and Oceania [4].

The incidence and prevalence of MS have been investigated in numerous studies, and various results have been reported in this regard [3–7]. The prevalence of MS is also different in various provinces of Iran [8]. The pertinent studies suggested that the occurrence of MS increased with the distance from the equator [9]. In this regard, the findings have revealed that while Africa and Asia have the lowest prevalence of MS, Northern Europe has reported the highest prevalence of MS [10]. Kurtzke [11] has divided different regions of the world into three categories of low (under 5 cases per 100,000 people), medium (5–25 cases per 100,000 people), and high (over 30 cases per 100,000 people) frequency regions in terms of MS incidence. Based on Kurtzke [11], Tehran is considered as a high frequency region for MS.

Previous studies have been conducted to estimate the incidence and prevalence of MS in Iran, and some of the mentioned studies have only investigated the familial MS [6, 12]. A recent study addressed the occurrence of MS in Tehran by examining the pertinent data obtained from 1991 to 2014 [7] and also another published study in Isfahan Province in 2014 was very brief. Therefore, considering the significant time trend in MS occurrence and the remarkable geographical variations in its incidence in Iran [8], further studies are required to be conducted to shed more light on the issue.

According to Hosseinzadeh et al. [8] and Kurtzke [11], Tehran is considered as one of the high-frequency provinces for MS. As the frequency of MS in Iran is high and varies over time, the present study aimed at investigating the longitudinal prevalence of MS in Tehran, Iran.

## Methods

The present study was conducted addressing the population of Tehran, the capital of Iran. *According to* the *2016 population census* conducted in Iran, the population of Tehran was 13,267,637, of which 6,673,672 (50.3%) and 6,593,965 (49.7%) were male and female, respectively.

This population-based study was conducted in Tehran province of Iran based on the annual report data provided by the Iranian MS Society (IMSS) registry system from 1st April 1999 to 31st December 2018. The registration program as well as its validity and reliability has been described in the previous study [13]. Briefly, the Iranian MS Society, established in 1999, is the single registry center in Tehran and comprehensively registers baseline characteristics including patient identification, family history of MS, diagnosis, disease course, disability status, and medications. The required data set was determined according to 27 experts’ opinions. Neurologists confirmed the disease diagnosis using the latest version of the McDonald criteria [14].

As IMSS provides extensive facilities for its members, all patients were encouraged by neurologists to refer to the IMSS to register and receive tracking code for receiving treatment and care services. The IMSS membership card must be activated by the patients every 5-year to keep their presence active. To design the present population-based cross-sectional study, it was tried to cover the most important epidemiological variables, which were related to MS recurrence at the individual level. The examined variables included sex, birth date (age), age at disease onset, and date of diagnosis.

A trained interviewer explained the objectives of the MS registry in IMSS to all patients. An informed written consent was obtained from all patients.

All patient data was collected from the data registry and consent was obtained when the data was originally collected and this study was approved by institutional review board of Tehran University of Medical Sciences, Tehran, Iran (Code: IR.TUMS.NI.REC.1398.009).

### Statistical analyses

Categorical and continuous data was presented as percentages and means (Standard Deviation (S.D.)). To calculate the prevalence of MS, direct standardization was used to adjust the effect of patients’ age using the world standard population [15].

The MS prevalence estimate was calculated using the population data obtained from the Statistical Centre of Iran. Age-standardized prevalence (ASP) was calculated per 100,000 people by “*dstdize*” command in Stata software. The Poisson regression model was used to estimate the significance of change over time for total prevalence, sex-specific prevalence, and sex ratio of MS. All analyses were performed using Stata software, Version 14 (Stata Corp, College Station, TX).

To calculate the prevalence, the target population was determined using the 2006, 2011 and 2016 census of the Statistical Center of Iran; otherwise, the exponential formula was used to estimate the population as follows:

$$ {\mathrm{P}}_{\mathrm{T}}={\mathrm{P}}_0\ {\left(1+\mathrm{r}\right)}^{\mathrm{t}} $$

where P_T_ is the population to be estimated (population at time T), P_0_ stands for the population at time zero, r is the growth rate that was considered as 1.44 [16] for 2007–2010 and 1.72 [17] for 2012–2015 and 2017–2018), and t is the elapsed time from time zero in the mentioned years.

## Results

### Age at disease onset and its trends

In the present population-based MS registry, 21,580 cases of MS were registered and included in the analysis. 5393 (24.99%) and 16,187 (75.01%) of the involved participants were male and female, respectively. The mean age of MS onset was 28.8 years (S.D: 8.7, ranged from 3 to 77 years). The mean age of males (29.5 years, S.D.: 8.9) was significantly (*p* < 0.001) higher than that of females (28.6 years, S.D.: 8.6). The mean age of MS onset in males and females in 2018 was 30.9 (S.D: 8.3) and 31.8 (S.D: 8.9) years, respectively. 13.4% of MS cases were familial MS patients. Demographic characteristics of MS cases are presented in Table 1.

**Table 1:** Demographic characteristics of MS cases at the onset of the disease in Tehran, Iran

Variables	N (%)
Sex	
Male	5393 (24.99)
Female	16,187 (75.01)
Age	
≤ 18	2023 (9.7)
19–24	5098 (24.7)
25–29	4807 (23.3)
30–34	3704 (18.0)
35–39	2395 (11.6)
40–44	1518 (7.4)
≥ 45	1091 (5.3)
Familial MS	
Yes	2815 (13.4)
No	18,145 (86.6)

### Age-standardized prevalence of MS

In Table 2, the crude and ASP of MS were reported according to patients’ sex. The ASP of MS increased from 73.7 (95%CI: 72.1–75.2) per 100,000 people in 2006 to 137.6 (95% CI: 135.7–139.5) per 100,000 people in 2018. The ASP of MS in 2018 was estimated to be 67.9 (95%CI: 66.0–69.8) and 207.3 (95%CI: 204.0–210.7) per 100,000 people among males and females, respectively. The results of Poisson regression model suggested that there was a significant change over time for the total prevalence (*p* = 0.001), prevalence among males (p = 0.001), and prevalence among females (*P* = 0.001).

**Table 2:** The crude and age standardized prevalence of MS in Tehran, Iran during 2006–2018

Year	Female				Male				Total			
	Population	Count	Prevalence*	ASP^**†**^ (95%CI)	Population	Count	Prevalence*	ASP^**†**^ (95%CI)	Population	Count	Prevalence*	ASP^**†**^ (95%CI)
2006	5,513,872	6848	124.2	115.4 (112.6–118.2)	5,831,418	2146	36.8	33.9 (32.4–25.3)	11,345,290	8994	79.3	73.7 (72.1–75.2)
2007	5,593,267	7633	136.5	126.7 (123.8–129.6)	5,915,395	2397	40.5	37.2 (35.7–38.7)	11,508,662	10,030	87.2	80.9 (79.3–82.5)
2008	5,673,810	8447	148.9	137.9 (134.9–141.0)	6,000,577	2665	44.4	40.9 (39.3–42.5)	11,674,387	11,112	95.2	88.3 (86.6–90.0)
2009	5,755,513	9232	160.4	148.3 (145.2–151.4)	6,086,985	2930	48.1	44.3 (42.7–45.9)	11,842,498	12,162	102.7	95.1 (93.4–96.8)
2010	5,838,393	10,160	174.0	160.6 (157.4–163.8)	6,174,637	3198	51.8	47.7 (46.0–49.3)	12,013,030	13,358	111.2	102.8 (101.0–104.6)
2011	6,045,398	11,187	185.1	170.2 (167.0–173.4)	6,137,993	3513	57.2	52.4 (50.6–54.2)	12,183,391	14,700	120.7	111.2 (109.3–113.0)
2012	6,149,379	12,028	195.6	179.4 (176.1–182.7)	6,243,566	3780	60.6	55.3 (53.5–57.1)	12,392,945	15,808	127.6	117.2 (115.4–119.1)
2013	6,255,149	12,867	205.7	188.2 (184.9–191.5)	6,350,955	4083	64.3	58.7 (56.8–60.5)	12,606,104	16,950	134.5	123.3 (121.4–125.2)
2014	6,362,737	13,583	213.5	194.9 (191.5–198.2)	6,460,192	4357	67.5	61.4 (59.6–63.3)	12,822,929	17,940	139.9	128.0 (126.1–129.9)
2015	6,472,176	14,211	219.6	200.0 (196.7–203.4)	6,571,307	4591	69.9	63.6 (61.7–65.4)	13,043,483	18,802	144.2	131.6 (129.7–133.6)
2016	6,593,965	14,875	225.6	205.0 (201.7–208.4)	6,673,672	4828	72.3	65.7 (63.8–67.6)	13,267,637	19,703	148.5	135.3 (133.4–137.3)
2017	6,693,000	15,304	228.7	207.6 (204.2–210.9)	6,768,000	5037	74.4	67.5 (65.6–69.4)	13,461,000	20,341	151.1	137.5 (135.6–139.5)
2018	6,784,000	15,505	228.6	207.3 (204.0–210.7)	6,853,000	5131	74.9	67.9 (66.0–69.8)	13,636,000	20,636	151.3	137.6 (135.7–139.5)

^*^Crude Prevalence (Per 100,000)

^**†**^Age Standardized Prevalence (Per 100,000)

The results showed that the prevalence of MS among females was significantly higher than that of males at all study years. Sex-specific prevalence data (count, population, prevalence) over the study period is show in Table 2.

### Sex ratio trends

**Fig. 1:**
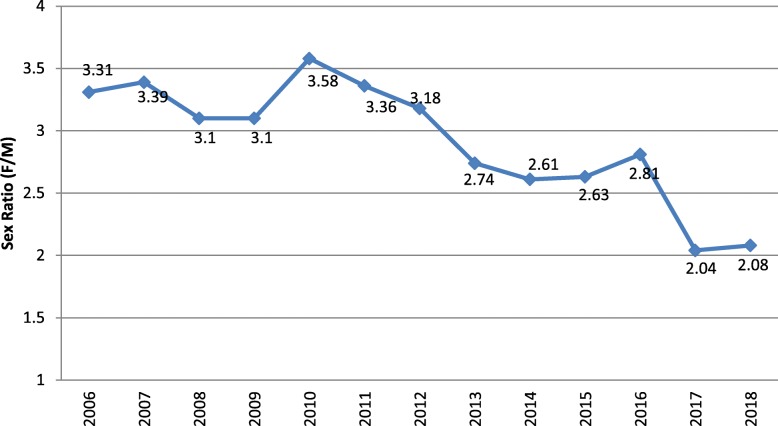
The age-standardized sex ratio (female to male) of MS, Tehran, Iran, 2006–2018

The sex ratios over the 13-year study period are displayed in Fig. 1. The sex ratio declined from 3.7 (in 2010) to 2.06 (in 2017) and had a decreasing trend. The results of Poisson regression model revealed that there was a significant change over time for sex ratio (*p* = 0.001).

## Discussion

The current study examined the trend of MS prevalence in Tehran, the capital of Iran, from 2006 to 2018. The results of this study suggested that the prevalence of MS in Tehran is increasing, and the unadjusted prevalence has increased from 79.3 cases per 100,000 people in 2006 to 151.7 cases per 100,000 people in 2018. Moreover, the age-standardized prevalence has increased from 73.7 cases per 100,000 people in 2006 to 137.6 cases per 100,000 people in 2018.

Similar studies have been conducted in countries surrounding Iran, countries in the Middle East, and Arab countries; however, it is important to note that as Iranians are identified as Persian, the heredity and genetic structure of Iranian population differ from those of the mentioned countries. Other studies have also been conducted in various parts of Iran and have indicated that the pattern of MS disease in Tehran may be different from that of other parts of the country. The observed pattern can be attributed to a number of differences between Tehran and other cities including the higher level of air pollution in Tehran [18].

Therefore, according to the global divisions [19], Tehran should be considered as a region with a high MS prevalence (prevalence ≥30/100,000). Moreover, Tehran has the highest MS prevalence in Asia and among the Middle Eastern countries [18]. In addition, the results revealed that the time trend of age-standardized MS female-to-male ratio in recent years has been declining to 2.14 in 2018.

A study conducted by Wallin MT et al. [3] in the United States in 2010 revealed that the prevalence of MS in the population aged over 18 years old was 309.2 per 100,000 people over 10 years. Moreover, the highest prevalence was reported for the 55–64 age group. As compared with the findings of the current study, in which most cases were observed in the age group below the age of 40 years, Wallin MT et al.’s study has a higher prevalence and a different age distribution. When coupled with prior estimates of the MS prevalence in Iran, it seems that there has been a relative increase in this regard over the last decades. In Eskandarieh et al.’s study [7], the MS prevalence in Tehran was 101.39 per 100,000 people in 2014, and the age-adjusted prevalence were 134 and 42.5 for females and males, respectively. In another review study, the MS prevalence in Iran was 85.8 per 100,000 people in 2013 [18].

According to Hosseinzadeh et al. [8], Tehran is considered as one of the high-frequency regions for MS. The high MS frequency in Tehran may be related to the rate of urbanization [20], the social and economic status [21, 22], and air pollution [23–25]. In addition, better access to health services can play an important role and in turn lead to better diagnosis and case registration [26].

Tehran has been recognized as one of the most polluted cities in the world [27]. Although some studies have not reported a significant association between MS and air pollution markers such as PM_2.5_, NO_2_, and O_3_ [28], several studies [23–25] have shown some evidences that air pollution can have a significant relationship with the incidence and recurrence of MS. Air pollution leads to deficiency of vitamin D, production of excessive free radical, expression of inflammatory factors, induction of chronic inflammation, dysfunction of mitochondrial, and increase of oxidative stress, all of which can be linked to MS incidence and recurrence [24, 25]. Therefore, as previous studies have shown [29], it can be hypothesized that a portion of the high prevalence of MS in Tehran may be attributable to the air pollution. To establish causality, it is recommended to compare different geographic regions with different pollution levels in terms of MS occurrence.

One finding presented in most of the pertinent studies is that women as compared with men are more susceptible to MS [30, 31]. In a study addressing a relatively large sample of Canadian MS patients, sex ratio was estimated to be 3.2, which was so similar to the findings of the current study [30]. Moreover, the sex ratio has been reported to be 3.06 in Eskandarieh et al.’s study [6]. A study conducted in Turkey [32] revealed that the prevalence of MS in Karabük and Akçakoca were 95.9 and 46.1 per 100,000 people, respectively [33]. In addition, the mentioned study indicated that the prevalence of MS in the Middle East and North Africa was 51.52 cases per 100,000 people [33]. The estimated prevalence in the current study revealed that MS is more prevalent in Tehran as compared with other cities in the Middle East countries.

It should be noted that the trend of sex ratio in the present study was rather declining from 3.20 in 2006 to 2.14 in 2018. Some reasons can be provided to justify the increase of MS incidence among males in Iran. First, a larger percentage of males than females are in the workforce, mainly in urban areas (61.3% vs. 11.6%) [34] and therefore they are more profoundly affected by the related stressors in the workplaces. Second, males are more exposed to air pollution because of outdoor work.

The obtained trend in the current study using the extracted data up to 2010 is in agreement with the world global trend, which indicates the increasing sex ratio in MS patients. However, the findings of the study examining the extracted data over recent years have demonstrated descending sex ratio changes, which are consistent with the findings of Norway, Tasmania, and Sweden researches. The mentioned studies have indicated the stable sex ratio changes and did not support the former ascending trend [30, 35, 36]. However, some studies have reported an increase in this trend [37, 38].

In terms of age groups, nearly 60% of cases in the current study were diagnosed with MS before the age of 30 years. Other similar studies have also shown a higher incidence rate of MS at an early age [7, 39]. The disease onset in the early age leads to an increase in the number of years lived with disability (YLDs) and eventually to disability-adjusted life years (DALYs) [4, 40, 41].

The present study had some limitations and strengths. Considering that Tehran is a large province in terms of both the population and geographical area, and MS registration is elective, the MS registration may be incomplete and some cases may not be registered. Therefore, the obtained prevalence may be underestimated. In addition, it should be highlighted that as IMSS registration facilitates provide health care services for patients, the mentioned point can be regarded as a strong inducement that may facilitate the registration and retention processes and thus be considered as study strength.

## Conclusion

The findings of this study suggested that the prevalence of MS in Tehran province is relatively high, and disease occurrence is more common in groups below 40 years of age as compared with the older age-range groups. The results suggested that the prevalence of MS was higher in women, and the trend of female-to-male sex ratio has been declining over the studied period.

## Data Availability

The data sets used and analyzed during the study are available from the corresponding author on reasonable request.

## Abbreviations

MS: Multiple Sclerosis
IMSS: Iranian MS Society
CI: Confidence Interval
S.D.: Standard Deviation
ASP: Age-Standardized Prevalence
